# Association of Circulating Trimethylamine *N*-Oxide and Its Dietary Determinants with the Risk of Kidney Graft Failure: Results of the TransplantLines Cohort Study

**DOI:** 10.3390/nu13010262

**Published:** 2021-01-18

**Authors:** Jose L. Flores-Guerrero, Maryse C. J. Osté, Paula B. Baraldi, Margery A. Connelly, Erwin Garcia, Gerjan Navis, Stephan J. L. Bakker, Robin P. F. Dullaart

**Affiliations:** 1 Department of Internal Medicine, Division of Nephrology, University of Groningen, University Medical Center Groningen, 9713 GZ Groningen, The Netherlands; m.c.j.oste@umcg.nl (M.C.J.O.); p.barreto.baraldi@umcg.nl (P.B.B.); g.j.navis@umcg.nl (G.N.); s.j.l.bakker@umcg.nl (S.J.L.B.); 2 Laboratory Corporation of America Holdings (LabCorp), Morrisville, NC 27560, USA; connem5@labcorp.com (M.A.C.); garce14@labcorp.com (E.G.); 3 Department of Internal Medicine, Division of Endocrinology, University of Groningen, University Medical Center Groningen, 9713 GZ Groningen, The Netherlands; dull.fam@12move.nl

**Keywords:** trimethylamine-*N*-oxide (TMAO), kidney transplantation, diet, net benefit

## Abstract

Background. Due to the critical shortage of kidneys for transplantation, the identification of modifiable factors related to graft failure is highly desirable. The role of trimethylamine-*N*-oxide (TMAO) in graft failure remains undetermined. Here, we investigated the clinical utility of TMAO and its dietary determinants for graft failure prediction in renal transplant recipients (RTRs). Methods. We included 448 RTRs who participated in the TransplantLines Cohort Study. Cox proportional-hazards regression analyses were performed to study the association of plasma TMAO with graft failure. Net Benefit, which is a decision analysis method, was performed to evaluate the clinical utility of TMAO and dietary information in the prediction of graft failure. Results. Among RTRs (age 52.7 ± 13.1 years; 53% males), the baseline median TMAO was 5.6 (3.0–10.2) µmol/L. In multivariable regression analysis, the most important dietary determinants of TMAO were egg intake (Std. β = 0.09 [95%CI, 0.01; 0.18]; *p* = 0.03), fiber intake (Std. β = −0.14 [95%CI, −0.22, −0.05]; *p* = 0.002), and fish and seafood intake (Std. β = 0.12 [95%CI, 0.03,0.21]; *p* = 0.01). After a median follow-up of 5.3 (4.5–6.0) years, graft failure was observed in 58 subjects. TMAO was associated with an increased risk of graft failure, independent of age, sex, the body mass index (BMI), blood pressure, lipids, albuminuria, and the Estimated Glomerular Filtration Rate (eGFR) (Hazard Ratio per 1-SD increase of TMAO, 1.62 (95% confidence interval (CI): 1.22; 2.14, *p* < 0.001)). A TMAO and dietary enhanced prediction model offered approximately double the Net Benefit compared to a previously reported, validated prediction model for future graft failure, allowing the detection of 21 RTRs per 100 RTRs tested, with no false positives versus 10 RTRs, respectively. Conclusions. A predictive model for graft failure, enriched with TMAO and its dietary determinants, yielded a higher Net Benefit compared with an already validated model. This study suggests that TMAO and its dietary determinants are associated with an increased risk of graft failure and that it is clinically meaningful.

## 1. Introduction

Renal transplantation is the treatment of choice for patients with end-stage kidney disease, being superior to dialysis in terms of quality of life, long-term survival, and healthcare costs [1]. Unfortunately, worldwide, there is a massive shortage of organs. For example, in the United States of America, approximately 84% of patients with end-stage kidney disease will not receive a transplant and their prognosis is reduced to an average of 5-year survival on dialysis before premature death [2].

Due to the scarcity of organs, approaches for lengthening graft and patient survival are highly desirable [3]. Over the last few decades, there has been a remarkable improvement in 1-year graft survival; nonetheless, long-term survival still needs to be improved [3,4]. The investigation of biomarkers that reflect pathophysiological changes in the interplay of the kidney with other organs, such as crosstalk between the gut microbiome and the kidney, could be of utility in the development of future interventions for preserving the graft function.

The evaluation of the above-mentioned biomarkers should take into account the clinical utility of the biomarkers, in addition to their predictive accuracy. The application of decision theory analytical methods, i.e., the calculation of Net Benefit, has been proposed as an alternative for evaluating the clinical value of biomarkers and predictive models [5,6].

Trimethylamine-*N*-oxide (TMAO) is a methylamine osmolyte [7] that has gained attention due to its potential role in the development of heart [8,9] and kidney disease [10], and mortality [11]. TMAO is a product of the gut microbiome–host metabolism of dietary components such as choline and L-carnitine [12,13]. Its metabolism includes the production of trimethylamine (TMA) by the gut microbiota, subsequent conversion to TMAO by host liver flavin monooxygenase 3, and the clearance of circulating TMAO by the kidneys [14].

Despite the fact that some studies have identified an association between plasma concentrations of TMAO and adverse cardiovascular outcomes in patients with chronic kidney disease [15,16] and in hemodialysis patients [17], the potential role of these osmolytes in graft survival in renal transplant recipients is still unknown. Considering that modification of the gut microbiome by dietary intervention may enhance survival of the allograft, as observed in a rodent kidney transplant model [18], and the fact that TMAO may contribute to renal fibrosis [19], the aim of the present study was to evaluate the potential association of TMAO and its dietary determinants with the risk of graft failure in renal transplant recipients (RTRs) and its clinical value.

## 2. Materials and Methods

### 2.1. Study Population and Data Collection

In this study, stable adult RTRs (≥18 years old) with a functioning graft at least one year after transplantation (i.e., on maintenance immunosuppression and with a stable renal function) were included. Briefly, between November 2008 and May 2011, patients who visited the outpatient clinic of the University Medical Center Groningen were invited to participate. Subjects with malignancies, opportunistic infections, or addictions were excluded. Furthermore, participants with missing data on TMAO were excluded, resulting in 448 renal transplant recipients. The protocol for the present study was approved by the local ethics committee of the University Medical Center Groningen (METc 2008/186), and all procedures were conducted according to the Declaration of Helsinki [20]. This report follows the Strengthening the Reporting of Observational Studies in Epidemiology (STROBE) reporting guideline (Supplemental Table S1).

During a morning visit to the outpatient clinic, all baseline data were collected as previously described [21]. The height and weight were measured with the participants standing without shoes and heavy outer garments. The body mass index (BMI) was calculated by dividing the weight in kilograms by the height in meters squared. The systolic and diastolic blood pressure and heart rate were measured every minute for 15 min in a half-sitting position using a semi-automatic device (Dinamap^®^1846; Critikon, Tampa, FL, USA). The average of the last three measurements was taken as the blood pressure value. Information on medication was derived from patient records. Information on smoking behavior was obtained by a questionnaire.

### 2.2. Dietary Intake Assessment

Dietary intake was assessed with a validated semi-quantitative food frequency questionnaire (FFQ) developed and updated by Wageningen University (Wageningen, The Netherlands) [22]. The intake of 177 food items during the previous month was obtained, taking seasonal variations into account. For each item, the frequency was registered daily, weekly, and monthly. In this study, only measurements of daily intake were considered. The FFQ was self-administered and its completeness was checked by a trained researcher on the day of the visit to the outpatient clinic. Inconsistent answers were verified with the patients. The results of the FFQ were converted into the total energy (in kcal) per day by using the Dutch Food Composition Table (NEVO 2006). The FFQ was validated by comparing the protein intake of the FFQ with the protein intake calculated by the Maroni Equation, using urinary urea excretion values [23].

### 2.3. Laboratory Measurements

Blood samples were taken after an 8–12 h overnight fasting period in the morning after the completion of 24 h urine collection. Serum creatinine was determined using a modified version of the Jaffé method (MEGA AU 510, Merck Diagnostica, Darmstadt, Germany). TMAO concentrations were measured in EDTA anticoagulated plasma samples using a Vantera^®^ Clinical Analyzer (LabCorp, Morrisville, NC, USA), which is a fully automated, high-throughput, 400 MHz proton (1H) nuclear magnetic resonance (NMR) spectroscopy platform. Plasma samples were prepared onboard the instrument and automatically delivered to the flow probe in the NMR spectrometer’s magnetic field. TMAO concentrations were quantified from one-dimensional (1D) proton (1H) Carr–Purcell–Meiboom–Gill (CPMG) spectra by means of deconvolution assays, as previously described [24]. The TMAO assay has intra- and inter-assay coefficients of variation (CV%) that range from 4.3–10.3% and 9.8–14.5%, respectively, and a limit of quantitation of 3.3 μM.

The Estimated Glomerular Filtration Rate (eGFR) was calculated using the serum creatinine-based Chronic Kidney Disease Epidemiology Collaboration equation [25]. The Chronic Kidney Disease (CKD) stage was determined according to the latest nomenclature from Kidney Disease: Improving Global Outcomes (KDIGO) Consensus Conference [26] (normal eGFR (G1): ≥90 mL/min/1.73 m^2^, mildly decreased (G2): 60–89, mildly to moderately decreased (G3a): 45–59, moderately to severely decreased (G3b): 30–44, severely decreased (G4): 15–29, and kidney failure (G5): <15).

### 2.4. Clinical Endpoint

The primary outcome of this study—graft failure—was defined as re-transplantation or return to dialysis and was censored for death. The endpoint was recorded by a qualified physician until September 2015, with no loss to follow-up.

### 2.5. Statistical Analysis 

Normally distributed data were presented as the mean and standard deviation, whereas skewed data were expressed as the median and interquartile range. Categorical data were presented as the number and percentage. Linear trends across TMAO tertiles were determined using ANOVA for normally distributed data, the Kruskal–Wallis test for skewed distributed data, and the χ^2^ test for categorical variables. Skewed data were log-transformed when appropriate. Baseline associations between characteristics and TMAO concentrations were analyzed through univariable regression analysis and backward stepwise regression analysis, which involves a sequence of automated steps in which the least statistically significant explanatory variables are removed from a full model, until the most parsimonious model is achieved. In order to identify the risk of multicollinearity in the multivariable regression analysis, Variance-Inflation Factors were calculated. A high risk of multicollinearity was considered present if the calculated Variance-Inflation Factor (VIF) was >5 [27].

For the prospective analysis, we plotted cumulative Kaplan–Meier curves for the risk of graft failure during follow-up, according to the tertiles of TMAO. Time-to-event Cox proportional hazards models were used to compute hazard ratios (HRs) and 95% CI of the graft failure risk among the 448 participants. HRs were calculated in models adjusted for age, sex, pre-emptive transplantation, donor type, immunosuppressive medication, HLA (human leukocyte antigen)-A,-B and HLA-DR broad antigen mismatch, a history of type 2 diabetes (T2D), tobacco and alcohol consumption, BMI, systolic blood pressure, glucose, total cholesterol, high-density lipoprotein cholesterol (HDL-cholesterol), triglycerides, insulin, urinary albumin excretion (UAE), CKD stage [26] (normal eGFR (G1): ≥90 mL/min/1.73 m^2^, mildly decreased (G2): 60–89, mildly to moderately decreased (G3a): 45–59, moderately to severely decreased (G3b): 30–44, severely decreased (G4): 15–29, and kidney failure (G5): <15), vegetable intake, meat intake, and fish and seafood intake. The Cox proportional hazard assumption was tested through the evaluation of independence between scaled Schoenfeld residuals with time for each variable and for every model as a whole; this assumption was met, with no indication of a violation [28].

To determine whether the model with a higher HR can improve the predictive ability of an independent multicenter validated predictive model for kidney graft failure that includes age, sex, serum albumin, the urine albumin-creatinine ratio, and eGFR [29], the net reclassification improvement (NRI) [30] was calculated for an enhanced model that included TMAO and its dietary determinants, according to the cross-sectional relationships and previous reports in the literature [13,31,32,33,34], i.e., fish and seafood, red meat, and eggs. In order to avoid bias, for the computation of NRI, four predefined risk categories for kidney graft failure previously described in the literature were used: Low (<5%); medium low (5% to 10%); medium high (11% to 20); and high (>20%) [29].

Brier scores were calculated in order to assess the difference between the predicted probability of graft failure development and the cases of actual outcome development [35]. The Brier scores of two predictive models for kidney graft failure were compared: A model already validated [29] and our model enhanced with TMAO and dietary information.

Since Brier scores could not properly assess the clinical utility of some prediction models [36], and considering that traditional measures, such as sensitivity, specificity, or area under the curve, correspond to statistical abstractions which could not provide direct information about the clinical value of biomarkers or prediction models, we decided to calculate the Net Benefit, which is a decision-analytic measure that can overcome such limitations [5]. Therefore, the clinical utility of enhancing the previously validated model with TMAO and dietary information was further assessed by calculating the Standardized Net Benefit and plotting decision curves for both models after 1000 bootstraps.

Briefly, Net Benefit is a type of decision analysis method in which an exchange rate is calculated for the benefit and harm of a prognostic model. In the clinical research of prediction biomarkers and models, the above-mentioned exchange rate is related to the probability of a patient being classified as a developer of the disease. Net Benefit informs about the proportion of “net” true positives. The “net” can be understood as the observed number of true positives corrected for the observed proportion of false positives and weighted by the odds of the risk threshold [37]. Further details about the methods and rationality of decision theory in clinical research can be found elsewhere [6,38].

All statistical analyses were performed with R language for statistical computing software [39], v. 4.0.2, R Foundation for Statistical Computing, Vienna, Austria.

## 3. Results

### 3.1. Baseline Characteristics

Of the 632 participants that were enrolled in the prospective cohort, 448 subjects with available measurements of TMAO were included in the current study. Among the study participants, 238 (53.1%) were men and the mean age of the population was 54.9 ± 13.1 years. The median (IQR) plasma total TMAO concentration was 5.65 (3.1–11.2) μM. Participant characteristics at the baseline are shown in Table 1. Participants within the highest tertile of TMAO plasma concentrations (>8.07 μmol/L) were more likely to be older; have lower concentrations of total and HDL-cholesterol, decreased eGFR, and higher urinary albumin excretion; and belong to an advanced CKD stage. Those patients reported a higher consumption of fish and seafood; less frequently received renal allografts from living donors; and were more frequently taking cyclosporine, azathioprine, mycophenolic acid, and diuretic medication.

### 3.2. Cross-Sectional Analyses

The association of the concentration of TMAO with baseline characteristics was evaluated with both univariable and multivariable linear regression analyses. In the univariable analyses, HDL-C (Std. β = −0.12 [95%CI, −0.21, −0.02]; *p* = 0.01), eGFR (Std. β = −0.29 [95%CI, −0.38, −0.20]; *p* < 0.001), stage 4 CKD (Std. β = 0.88 [95%CI, 0.29, 1.15]; *p* = 0.003), stage 5 CKD (Std. β = 1.00 [95%CI, 0.02, 1.90]; *p* = 0.003), fish and seafood intake (Std. β = 0.15 [95%CI, 0.06, 0.24]; *p* < 0.001), a living donor (Std. β = −0.24 [95%CI, −0.43, −0.05]; *p* = 0.02), and tacrolimus medication (Std. β = 0.31 [95%CI, 0.08, 0.54]; *p =* 0.009) were significantly associated with plasma concentrations of TMAO (Table 2). In a multivariable model, without the risk of multicollinearity (VIF < 5), TMAO remained independently associated with systolic blood pressure (SBP) (Std. β = 0.15 [95%CI, 0.03, 0.27]; *p* = 0.01), diastolic blood pressure (DBP) (Std. β = −0.13 [95%CI, −0.25,−0.02]; *p* = 0.03), total cholesterol (TC) (Std. β = −0.15 [95%CI, −0.24,−0.06]; *p* = 0.01), eGFR (Std. β = −0.28 [95%CI, −0.36, −0.19]; *p* < 0.001), egg intake (Std. β = 0.09 [95%CI, 0.01, 0.18]; *p* = 0.03), fiber intake (Std. β = −0.14 [95%CI, −0.22, −0.05]; *p* = 0.002), and fish and seafood intake (Std. β = 0.12 [95%CI, 0.03,0.21]; *p* = 0.01) (Table 2). Notably, the distribution of the concentrations of TMAO in RTRs in the highest tertiles of fish and sea food intake differed among the participants with a normal and declined renal function. The median plasma concentration of TMAO in RTRs with eGFR > 60 mL/min * 1.73m^2^ was 3.04 (1.84–5.16), whereas the median for RTRs with eGFR < 60 mL/min * 1.73m^2^ was 12.82 (6.69–19.58), *p* < 0.001 (Supplemental Figure S1A and Supplemental Figure S1B). A similar effect modification was observed in RTRs in the highest tertiles of meat intake (Supplemental Figure S2A and Supplemental Figure S2B).

### 3.3. Longitudinal Analyses

**Figure 1:**
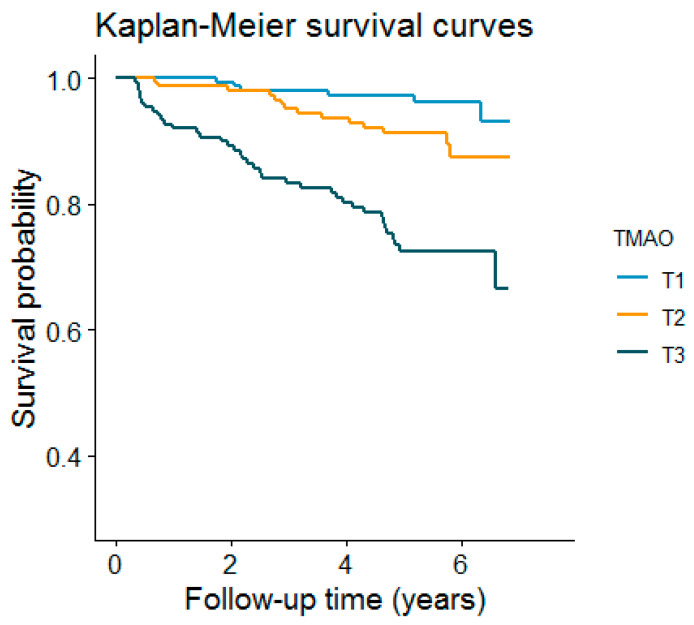
Kaplan–Meier plot for graft failure comparing tertiles of TMAO. (log-rank test, *p* value < 0.001).

After a median (IQR) follow-up of 5.3 (4.6–6.0) years, 58 (12.9%) RTRs developed graft failure. Kaplan–Meier curves for graft failure according to tertiles of the TMAO plasma concentration are presented in Figure 1. There was an increased risk of graft failure associated with the top tertile of TMAO concentrations (*p* for the log-rank test < 0.001).

In Cox regression analyses that examined the TMAO plasma concentration as a categorical variable with the first tertile as the reference group, the third tertile of the TMAO plasma concentration [8.07–154.3 μmol/L] was associated with an increased risk of graft failure, independent of age and sex (adjusted HR, 8.17 [95%CI, 3.44; 19.43]; *p* < 0.001, model 1, Table 3); pre-emptive transplantation, donor type (living or post mortem), T2D, smoking, alcohol consumption and immunosuppressive medication (tacrolimus, cyclosporine, and prednisolone), and HLA-A,-B and HLA-DR broad antigen mismatch (adjusted HR, 5.37 [95%CI, 2.15; 13.42]; *p* < 0.001, model 2, Table 3); BMI, systolic blood pressure, HDL-cholesterol, triglycerides, total cholesterol, UAE, and CKD stage (adjusted HR, 3.01 [1.10; 8.26]; *p* = 0.03, model 3, Table 3); and vegetable intake, meat intake, fish and seafood intake, and egg intake (adjusted HR, 9.15 [95%CI, 3.79; 22.04]; *p* < 0.001, model 4, Table 3).

The proportional hazards assumptions were not violated for any of the variables in the additive models. The interaction terms of the TMAO plasma concentration with age, sex, and eGFR with graft failure were not significant (*p* > 0.10 for each) when included in either the crude or the sex- and age-adjusted models.

Plasma concentrations of TMAO, when analyzed as HR per 1 log SD increase, were also associated with the risk of graft failure (Supplemental Figure S3). In Cox regression analyses that examined the TMAO plasma concentration as a continuous variable, the crude model displayed an HR of 2.22 ([95%CI, 1.73; 2.84]; *p* < 0.001 (Table 3)). The association was independent of adjustment for age and sex (adjusted HR, 2.20 [95%CI, 1.73; 2.80]; *p* < 0.001, model 1, Table 3); pre-emptive transplantation, donor type (living, post mortem), T2D, smoking, alcohol consumption and immunosuppressive medication (tacrolimus, cyclosporine, and prednisolone), and HLA-A,-B and HLA-DR broad antigen mismatch (adjusted HR, 2.27 [95%CI, 1.64; 3.14]; *p* < 0.001, model 2, Table 3); BMI, systolic blood pressure, HDL-cholesterol, triglycerides, total cholesterol, urinary albumin excretion, and CKD stage (adjusted HR, 1.39 [95%CI, 1.01; 1.91]; *p* = 0.04, model 3, Table 3); and vegetable intake, meat intake, fish and seafood intake, and egg intake (adjusted HR, 2.86 [95%CI, 2.13; 3.83]; *p* < 0.001, model 4, Table 3).

### 3.4. Clinical Utility of TMAO and Diet Assessment

The NRI of a previously validated model [29] that included age, sex, serum albumin, the urine albumin-creatinine ratio, and eGFR was compared with a model that included TMAO and its dietary determinants. Ten percent of the participants in the low-risk category were correctly reclassified, 48% of the participants in the medium-risk category were correctly reclassified, and 17% of the participants in the high-risk category were correctly reclassified with the addition of TMAO and dietary information to the risk model. The improvement in the division of participants into predicted risk categories was statistically significant, with an NRI of 0.35 ([95% CI, 0.08–0.62]; *p* = 0.01). The Brier Score of the updated model containing TMAO and dietary information showed a discrete, albeit insignificant, improvement from 0.0879 to 0.0857 (*p* > 0.5) (Supplemental Table S2).

**Figure 2:**
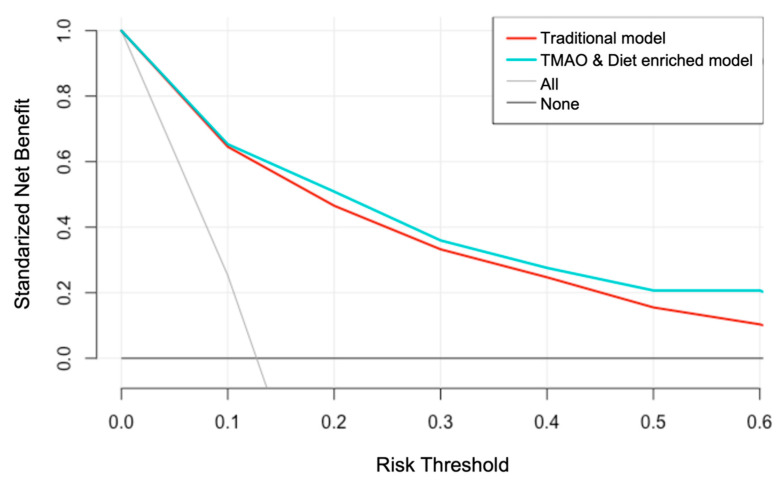
Decision curve for graft failure prediction. The model enriched with TMAO and diet information shows a superior clinical utility than the traditional risk model for graft failure.

The Net Benefit analysis demonstrated a higher clinical utility of the model containing TMAO and dietary information over the traditional model for graft failure risk prediction (Figure 2).

The TMAO and dietary enhanced model offered a greater Net Benefit, compared with the traditional model, for all the risk thresholds. The already validated model showed a Net Benefit of 0.466 at the 20% graft failure risk threshold, which is equivalent to detecting 46.6 RTR with future graft failure and suggesting no false positive results per 100 RTRs with future graft failure tested. At the same risk threshold, the TMAO and dietary enhanced model offered the greatest Net Benefit of 0.509, meaning that 51 RTRs with future graft failure could be detected per 100 RTRs tested, with no false positives. The TMAO and dietary enhanced model offered a double Net Benefit, compared with the traditional model at the 60% graft failure risk threshold, allowing the detection of 21 RTRs (per 100 RTRs tested with no false positives) with future graft failure. In contrast, the previously published model allowed the detection of 10 RTRs (per 100 RTRs tested with no false positives) (Supplemental Table S4).

## 4. Discussion

In this RTR cohort, the associations of plasma concentrations of TMAO with the risk of graft failure were investigated. Plasma concentrations of TMAO at the baseline were associated with reduced eGFR, and fish and seafood intake. The association of TMAO with an increased risk of graft failure remained significant when taking into account important risk factors, including the BMI, lipid profile, smoking, donor characteristics, diet, and renal function at the baseline.

Furthermore, the addition of circulating TMAO and diet information to an already validated predictive model of graft failures enhanced its predictive ability in terms of NRI, as well as Net Benefit. To the best of our knowledge, the present study is the first to extensively evaluate the clinical utility of circulating TMAO and diet information in the context of graft failure prediction in RTRs.

In this cohort study, the baseline renal function evaluated by means of eGFR was the variable with the strongest TMAO association (standardized regression coefficient (−0.28 [−0.37, −0.19], *p* < 0.001)). This result is in line with a previous study conducted in patients with end-stage renal disease where pre- and post-hemodialysis TMAO concentrations were measured, and showed a marked decline in TMAO concentrations, from 99.9 ± 31.9 to 41.3 ± 18.8 μM, after hemodialysis [40]. In relation to the dietary components, after multiple adjustment, fish and seafood intake combined was associated with TMAO plasma concentrations (standardized regression coefficient 0.12 [95%CI, 0.03,0.21], *p* = 0.01). These findings are explained by the role of fish and seafood as sources of TMAO, given the fact that such foods are the only source of TMAO that do not require intermediate metabolism [41]. Egg consumption displayed a similar regression coefficient with TMAO plasma concentrations (standardized regression coefficient 0.09 [95%CI, 0.01,0.18], *p* = 0.03). Such an association is in line with previous studies which have reported that an increased egg intake was associated with elevated concentrations of TMAO in both plasma and urine [42]. Moreover, the consumption of fiber was inversely associated with the plasma concentrations of TMAO, (standardized regression coefficient− 0.14 [−0.22, −0.05], *p* = 0.002). Notably, subjects in the highest tertile of TMAO consumed less vegetables and fruits and more eggs and fish and seafood than the average Dutch person (Supplemental Table S3).

Post-hemodialysis concentrations were closer to the values in the healthy control group (37.8 ± 20.4 μM). Notably, the prospective association of TMAO concentrations with graft failure was independent of CKD stage. The role of the gut microbiome in the context of chronic kidney disease has gained recent attention [43]. However, little is known about its role and clinical utility in graft survival after kidney transplantation. In a pilot study involving 26 RTRs, it was found that posttransplant diarrhea, which is associated with allograft failure [44] and affects one in five RTRs in the first year after kidney transplantation, was associated with gut microbiome dysbiosis, characterized by a reduction in the relative abundance of commensal bacterial taxa [45]. In this context, the positive association of TMAO with graft failure risk suggests a potential link between the gut microbiome and the patency of the graft in RTRs.

It is necessary to have reliable information about the clinical benefit of gut microbiome-related biomarkers and their modifiable determinants (i.e., diet) for the prediction of graft survival in RTRs. Due to the fact that traditional measures, such as the sensitivity, specificity, or area under the curve, correspond to statistical abstractions which cannot provide direct information about the clinical value of biomarkers or prediction models, the use of decision-analytic measures, such as Net Benefit, has been proposed [5].

Our results from Net Benefit analysis showed that the addition of TMAO measurements and dietary information increases the Net Benefit of an already validated graft failure predicted model [29]. The previous prediction model of 5-year graft failure was developed in Birmingham, the United Kingdom (*n* = 651, alive), and it was externally validated in independent cohorts from Tours, France (*n* = 736); Leeds, the United Kingdom (*n* = 787); and Halifax, Canada (*n* = 475). In our study, the clinical utility of the model was improved by the addition of TMAO measurements and dietary information, identifying more RTRs with future graft failure, without increasing the false positives cases. Such improvement is more marked in a threshold risk of graft failure < 40%. Interestingly, RTRs under such a threshold risk are those who obtain a greater benefit from more sensitive predictive biomarkers, given the fact that they have a better renal function at the baseline and presumably are those in which a dietary intervention may have a greater impact.

According to the authors who first introduced the decision curve analysis as a method to evaluate prediction models and diagnostic tests, in classical decision theory, the strategy with the highest expected utility should be chosen, irrespective of the size or statistical significance of the benefit [6]. Therefore, even a marginal improvement may have a clinical significance.

### Strengths and Limitations

This single-center study has several strengths. In our analysis, TMAO improved the reclassification of patients from a lower to a higher graft failure risk category. The performance of circulating TMAO is in line with the previously reported performance of urinary TMAO prediction of a prolonged duration of graft function in kidney transplant recipients. It is interesting to note that, in a relatively small study (*n* = 53), urinary TMAO adequately discriminated patients with a limited graft function from those with a prolonged graft function [46].

The results of the present study obtained from Net Benefit analysis are particularly important due to the fact that the variables that enriched our model, consisting of the TMAO concentrations and dietary information, do not involve any invasive, dangerous, or costly procedures when obtaining them. To the best of our knowledge, this is the first report to include such an approach in the context of graft survival analyses. Moreover, those variables, contrarily to other biomarkers (i.e., genetic risk scores), represent modifiable factors that can be translated into nutritional recommendations in order to improve graft survival in RTRs. However, randomized clinical trials are required to demonstrate that the implementation of TMAO-lowering diets can actually prolong graft function in RTRs.

Several limitations of the present study deserve to be mentioned. First, the present study was conducted in the north of the Netherlands, and mainly comprises individuals of Caucasian ancestry, which could limit the extrapolation of our findings. For instance, the previously validated model used to predict graft failure included ethnicity [29], which was not included in the present study, given the fact that all of the participants, except one, were of Caucasian ancestry. Moreover, another model used for graft loss prediction revealed the utility of assessing the type of medical insurance (Medicare or private) [47]. In our model, it was not possible to evaluate such a variable given the fact that, in The Netherlands, there is a universal healthcare system. In addition, the diet in this cohort was only assessed at the baseline, and therefore, we could not provide any inference about the changes or permanence of the dietary habits over the follow-up period. Finally, due the nature of the observational studies, the associations reported in the present study could not immediately be translated into causal relationships, and therefore, further research is needed.

## 5. Conclusions

In conclusion, this study of stable RTRs revealed that high concentrations of circulating TMAO are associated with a higher risk of graft failure, independent of other risk factors, such as an impaired renal function. Furthermore, this study is the first to provide an extensive evaluation of the clinical utility of TMAO and dietary information for the risk classification and prediction of graft failure in RTRs. Further investigation is needed to unravel the complexity underlying the relationship between circulating gut microbiome-derived metabolites such as TMAO and CKD.

## Figures and Tables

**Table 1:** Participant characteristics according to tertiles of the plasma concentration of trimethylamine-*N*-oxide (TMAO).

Characteristic	All (*n* = 448)	Tertile 1 (*n* = 147) (<3.93 μmol/L)	Tertile 2 (*n* = 149) (3.93 to 8.07 μmol/L)	Tertile 3 (*n* = 152) (>8.07 μmol/L)	*p*-Value
Men, *n* (%)	238 (53.1%)	70 (47.6%)	79 (53.0%)	89 (58.6%)	0.17
Age, year	52.71 (13.09)	50.32 (13.14)	53.43 (12.64)	54.32 (13.23)	0.02
BMI, kg/m^2^	26.41 (4.79)	26.47 (5.01)	26.14 (4.37)	26.63 (4.97)	0.67
SBP, mmHg	135.85 (17.56)	135.25 (15.58)	134.47 (17.55)	137.77 (19.24)	0.24
DBP, mmHg	81.86 (10.95)	82.35 (10.49)	81.50 (10.85)	81.73 (11.54)	0.79
Heart Rate, bpm	67.97 (11.67)	68.20 (11.58)	68.03 (11.63)	67.69 (11.86)	0.93
Glucose, mmol/L	5.20 (4.70, 5.90)	5.30 (4.80, 5.70)	5.15 (4.70, 5.90)	5.30 (4.70, 6.00)	0.69
TG, mmol/L	1.88 (0.94)	1.80 (0.91)	1.89 (1.01)	1.95 (0.89)	0.21
TC, mmol/L	5.12 (1.14)	5.18 (1.15)	5.11 (1.19)	5.07 (1.08)	0.72
HDL-C, mmol/L	1.37 (0.46)	1.47 (0.48)	1.35 (0.44)	1.31 (0.46)	0.01
HbA1c, %	5.80 (5.50, 6.10)	5.80 (5.45, 6.25)	5.70 (5.40, 6.00)	5.80 (5.50, 6.10)	0.32
TMAO, μmol/L	5.66 (3.08, 11.20)	2.41 (1.44, 3.06)	5.62 (4.82, 6.69)	15.09 (11.08, 22.11)	<0.001
eGFR, mL/min/1.73 m^2^	49.93 (19.38)	62.12 (16.70)	49.35 (15.58)	38.71 (18.23)	<0.001
CKD stage, *n* (%)					<0.001
G1	12 (2.7%)	8 (5.4%)	2 (1.3%)	2 (1.3%)	
G2	125 (27.9%)	72 (49.0%)	38 (25.5%)	15 (9.9%)	
G3a	115 (25.7%)	41 (27.9%)	47 (31.5%)	27 (17.8%)	
G3b	128 (28.6%)	25 (17.0%)	47 (31.5%)	56 (36.8%)	
G4	62 (13.8%)	1 (0.7%)	15 (10.1%)	46 (30.3%)	
G5	6 (1.3%)	0 (0.0%)	0 (0.0%)	6 (3.9%)	
UAE, mg/24 h	43.97 (11.75, 207.12)	37.70 (9.62, 177.82)	30.55 (8.48, 108.65)	90.03 (16.54, 384.67)	<0.001
Current smokers, *n* (%)	56 (13.2%)	15 (11.1%)	16 (11.3%)	25 (17.0%)	0.24
Alcohol consumption, g/day	2.20 (0.04, 10.47)	2.01 (0.03, 11.33)	2.49 (0.06, 9.65)	2.62 (0.03, 10.33)	0.94
Total energy intake, kcal/day	2138.61 (613.46)	2204.56 (558.62)	2137.56 (650.91)	2072.21 (626.80)	0.21
Egg intake, g/day	8.93 (4.46, 14.29)	8.93 (4.46, 14.29)	7.14 (4.46, 14.29)	8.93 (7.14, 14.29)	0.25
Vegetable intake, g/day	107.00 (71.50, 147.90)	110.71 (81.69, 144.21)	112.67 (72.81, 156.02)	95.75 (65.83, 140.15)	0.10
Fruit intake, g/day	105.43 (49.71, 191.53)	90.11 (39.63, 178.02)	123.79 (53.07, 212.64)	99.43 (52.71, 191.41)	0.25
Fiber intake, g/day	20.86 (16.32, 26.39)	21.07 (15.88, 27.15)	21.47 (17.93, 28.23)	19.95 (15.28, 25.29)	0.08
Fish and sea food intake, g/day	10.81 (4.42, 18.71)	9.94 (3.89, 17.07)	10.69 (4.68, 16.93)	14.87 (4.68, 22.96)	0.02
Meat intake, g/day	83.82 (60.95, 100.30)	84.06 (59.58, 102.90)	80.51 (64.76, 98.25)	84.60 (59.92, 99.49)	0.86
Transplant vintage, year	4.95 (1.59, 11.54)	4.88 (1.79, 10.26)	4.08 (1.31, 10.58)	6.14 (1.76, 14.43)	0.09
Preemptive, *n* (%)	69 (17.5%)	28 (20.9%)	26 (20.3%)	15 (11.3%)	0.07
Living donor, *n* (%)	158 (35.3%)	62 (42.2%)	57 (38.3%)	39 (25.7%)	0.007
HLA-A,-B broad antigen mismatch, *n* (%)					0.99
0	94 (22.8%)	29 (21.6%)	32 (22.7%)	33 (23.9%)	
1	97 (23.5%)	35 (26.1%)	33 (23.4%)	29 (21.0%)	
2	151 (36.6%)	48 (35.8%)	51 (36.2%)	52 (37.7%)	
3	46 (11.1%)	14 (10.4%)	16 (11.3%)	16 (11.6%)	
4	25 (6.1%)	8 (6.0%)	9 (6.4%)	8 (5.8%)	
HLA-DR broad antigen mismatch, *n* (%)					0.49
0	175 (42.6%)	60 (45.1%)	55 (39.0%)	60 (43.8%)	
1	192 (46.7%)	60 (45.1%)	66 (46.8%)	66 (48.2%)	
2	44 (10.7%)	13 (9.8%)	20 (14.2%)	11 (8.0%)	
Tacrolimus, *n* (%)	68 (17.2%)	17 (12.7%)	22 (17.2%)	29 (21.8%)	0.14
Cyclosporine, *n* (%)	173 (43.8%)	48 (35.8%)	60 (46.9%)	65 (48.9%)	0.07
Azathioprine, *n* (%)	72 (18.2%)	21 (15.7%)	17 (13.3%)	34 (25.6%)	0.02
Mycophenolic acid, *n* (%)	262 (66.3%)	98 (73.1%)	90 (70.3%)	74 (55.6%)	0.005
Prednisolone dose, mg/day	10.00 (7.50, 10.00)	10.00 (7.50, 10.00)	10.00 (7.50, 10.00)	10.00 (7.50, 10.00)	0.42
Statins, *n* (%)	233 (52.0%)	70 (47.6%)	73 (49.0%)	90 (59.2%)	0.09
Diuretics, *n* (%)	177 (39.5%)	47 (32.0%)	55 (36.9%)	75 (49.3%)	0.007

Abbreviations: BMI, body mass index; SBP, systolic blood pressure; DBP, diastolic blood pressure; BPM, beats per minute; TG, triglycerides; TC, total cholesterol; HDL-C, high-density lipoprotein cholesterol; eGFR, estimated glomerular filtration rate; UAE, urinary albumin excretion; HLA, human leukocyte antigen.

**Table 2:** Univariable and multivariable associations of baseline characteristics with plasma concentrations of TMAO in renal transplant recipients.

	Univariable Regression		Multivariable Regression	
Variable	β (95% CI)	*p*-Value	β (95% CI)	*p*-Value
Sex, male, yes	0.02 (−0.17, 0.21)	0.83	—	—
Age, year	0.03 (−0.06, 0.12)	0.54	—	—
BMI, kg/m^2^	0.06 (−0.04, 0.15)	0.24	—	—
SBP, mm Hg	0.06 (−0.03, 0.15)	0.21	0.15 (0.03, 0.27)	0.01
DBP, mm Hg	−0.05 (−0.14, 0.05)	0.32	−0.13 (−0.25, −0.02)	0.03
Heart rate, bpm	0.03 (−0.07, 0.12)	0.56	—	—
Glucose, mmol/L	0.02 (−0.08, 0.11)	0.72	—	—
TG, mmol/L	0.03 (−0.06, 0.12)	0.53	—	—
TC, mmol/L	−0.09 (−0.19, 0.00)	0.05	−0.15 (−0.24, −0.06)	0.001
HDL-C, mmol/L	−0.12 (−0.21, −0.02)	0.01	—	—
HbA1C, %	0.02 (−0.08, 0.11)	0.71	—	—
eGFR, mL/min/1.73 m^2^	−0.29 (−0.38, −0.20)	<0.001	−0.28 (−0.36, −0.19)	<0.001
CKD stage, yes				
G2	0.01 (−0.56, 0.57)	0.99	—	—
G3a	0.10 (−0.47, 0.67)	0.74	—	—
G3b	0.46 (−0.10, 1.00)	0.11	—	—
G4	0.88 (0.29, 1.50)	0.003	—	—
G5	1.00 (0.02, 1.90)	0.04	—	—
UAE, mg/24 h	0.08 (−0.01, 0.17)	0.08	—	—
Current smoking, yes	0.10(−0.19, 0.39)	0.49	—	—
Alcohol consumption, g/day	−0.04 (−0.13, 0.05)	0.39	—	—
Total energy intake, kcal/day	−0.07 (−0.16, 0.02)	0.12	—	—
Egg intake, g/day	0.03 (−0.06, 0.11)	0.57	0.09 (0.01, 0.18)	0.03
Vegetable intake, g/day	−0.07 (−0.16, 0.01)	0.10	—	—
Fruit intake, g/day	−0.04 (−0.13, 0.04)	0.33	—	—
Vegetable and Fruit intake, g/day	−0.07 (−0.16, 0.02)	0.13	—	—
Fiber intake, g/day	−0.08 (−0.17, 0.01)	0.06	−0.14 (−0.22, −0.05)	0.002
Fish and Seafood intake, g/day	0.15 (0.06, 0.24)	<0.001	0.12 (0.03,0.21)	0.01
Meat intake, g/day	−0.01 (−0.09, 0.08)	0.89	—	—
Transplant vintage, year	0.01 (−0.08, 0.10)	0.84	—	—
Preemptive, yes	−0.23 (−0.47, 0.00)	0.05	—	—
Living donor, yes	−0.24 (−0.43, −0.05)	0.02	−0.08 (−0.17, 0.00)	0.06
HLA-A,-B broad antigen mismatch, yes				
1	−0.12 (−0.41, 0.16)	0.39	—	—
2	0.04 (−0.22, 0.30)	0.75	—	—
3	0.05 (−0.31, 0.40)	0.80	—	—
4	−0.16 (−0.60, 0.29)	0.48	—	—
HLA-DR broad antigen mismatch, yes				
1	0.08 (−0.12, 0.29)	0.43	—	—
2	0.07 (−0.26, 0.41)	0.66	—	—
Tacrolimus, yes	0.31 (0.08, 0.54)	0.009	—	—
Cyclosporine, yes	−0.01 (−0.19, 0.17)	0.92	—	—
Azathioprine, yes	0.15 (−0.08, 0.38)	0.19	—	—
Mycophenolic acid, yes	−0.16 (−0.35, 0.03)	0.09	—	—
Prednisolone dose, mg/day	−0.01 (−0.10, 0.09)	0.89	—	—
Statins, yes	0.02 (−0.17, 0.21)	0.83	—	—
Diuretics, yes	0.23 (0.04, 0.42)	0.02	—	—

Standardized beta regression coefficients (95% confidence intervals) are shown. Abbreviations: β, standardized beta regression coefficient; BMI, body mass index; SBP, systolic blood pressure; DBP, diastolic blood pressure; BPM, beats per minute; TG, triglycerides; TC, total cholesterol; HDL-C, high-density lipoprotein cholesterol; eGFR, estimated glomerular filtration rate; UAE, urinary albumin excretion; HLA, human leukocyte antigen.

**Table 3:** Prospective associations of plasma TMAO with the risk of graft failure in renal transplant recipients.

	TMAO Per 1 SD Increment		Tertile 1 (0.21–3.92)	Tertile 2 (3.93–8.07)		Tertile 3 (8.07–154.3)	
Participants, *n*	448		147	149		152	
Events, *n*	58		6	14		38	
	HR [95% CI]	*p*-value		HR [95% CI]	*p*-value	HR [95% CI]	*p*-value
Crude Model	2.22 [1.73; 2.84]	<0.001	(ref)	2.41 [0.93; 6.27]	0.07	7.62 [3.22; 18.03]	<0.001
Model 1	2.20 [1.73; 2.80]	<0.001	(ref)	2.54 [0.97; 6.62]	0.06	8.17 [3.44; 19.43]	<0.001
Model 2	2.27 [1.64; 3.14]	<0.001	(ref)	1.73 [0.60; 4.95]	0.31	5.41 [2.17; 13.48]	<0.001
Model 3	1.39 [1.01; 1.91]	0.04	(ref)	2.06 [0.75; 5.70]	0.16	3.01 [1.10; 8.26]	0.03
Model 4	2.86 [2.13; 3.83]	<0.001	(ref)	2.02 [0.73; 5.57]	0.17	9.15 [3.79; 22.04]	<0.001

Data are presented as hazard ratios (HRs) with 95% confidence intervals (CIs) and p-values. Model 1: Model adjusted for age + sex. Model 2: Model 1 + pre-emptive transplantation + donor type + T2D + smoking + alcohol consumption + immunosuppressive medication (tacrolimus, cyclosporine, and prednisolone) + HLA-A, -B + HLA-DR. Model 3: Model 1 + BMI + Systolic Blood Pressure + HDL-C + TG + TC + UAE + CKD stage. Model 4: Model 1 + vegetable intake + meat intake + fish and seafood intake + egg intake. Abbreviations: T2D, type 2 diabetes; BMI, body mass index; HDL-C, high density lipoprotein cholesterol; TG, triglycerides; TC, total cholesterol; UAE, urine albumin excretion; eGFR, estimated glomerular filtration rate.

## Data Availability

The data presented in this study are available on request from the corresponding author. The data are not publicly available due to confidentiality agreement.

## Supplementary Materials

The following are available online at https://www.mdpi.com/2072-6643/13/1/262/s1: Supplemental Table S1. STROBE Statement—Checklist of items that should be included in reports of cohort studies; Supplemental Table S2. Diet components in subjects for the third TMAO tertile; Supplemental Table S3. Reclassification table of RTRs; Supplemental Table S4. Comparison of Standardized Net Benefits of two predictive models; Supplemental Figure S1. Plasma TMAO distribution by tertile of fish and seafood intake in RTRs with reduced eGFR; Supplemental Figure S2. Plasma TMAO distribution by tertile of meat intake in RTRs with reduced eGFR; Supplemental Figure S3. Association between TMAO and graft failure.
