# Supplementary material for: Association of Circulating Trimethylamine N-Oxide and Its Dietary Determinants with the Risk of Kidney Graft Failure: Results of the TransplantLines Cohort Study
Source: Nutrients. 2021 Jan 18;13(1):262. doi: 10.3390/nu13010262 (PMC7831477; doi:10.3390/nu13010262)
Supplement: Supplementary file 1 [file nutrients-13-00262-s001.pdf]

Supplemental Table 1. STROBE Statement—Checklist of items that should be included in reports of cohort studies

	Item No	Recommendation	Page No
Title and abstract	1	(a) Indicate the study's design with a commonly used term in the title or the abstract	1
		(b) Provide in the abstract an informative and balanced summary of what was done and what was found	4
Introduction			
Background/rationale	2	Explain the scientific background and rationale for the investigation being reported	5
Objectives	3	State specific objectives, including any prespecified hypotheses	6
Methods			
Study design	4	Present key elements of study design early in the paper	7
Setting	5	Describe the setting, locations, and relevant dates, including periods of recruitment, exposure, follow-up, and data collection	7
Participants	6	(a) Give the eligibility criteria, and the sources and methods of selection of participants. Describe methods of follow-up	7
		(b) For matched studies, give matching criteria and number of exposed and unexposed	
Variables	7	Clearly define all outcomes, exposures, predictors, potential confounders, and effect modifiers. Give diagnostic criteria, if applicable	9,10
Data sources/ measurement	8	For each variable of interest, give sources of data and details of methods of assessment (measurement). Describe comparability of assessment methods if there is more than one group	7,8
Bias	9	Describe any efforts to address potential sources of bias	7-10
Study size	10	Explain how the study size was arrived at	7
Quantitative variables	11	Explain how quantitative variables were handled in the analyses. If applicable, describe which groupings were chosen and why	9,10
Statistical methods	12	(a) Describe all statistical methods, including those used to control for confounding	9-11
		(b) Describe any methods used to examine subgroups and interactions	NA
		(c) Explain how missing data were addressed	7

		(d) If applicable, explain how loss to follow-up was addressed<br>(e) Describe any sensitivity analyses	
	Results		
Participants	13*	(a) Report numbers of individuals at each stage of study—eg numbers potentially eligible, examined for eligibility, confirmed eligible, included in the study, completing follow-up, and analysed	7
		(b) Give reasons for non-participation at each stage	7
		(c) Consider use of a flow diagram	
Descriptive data	14	(a) Give characteristics of study participants (eg demographic, clinical, social) and information on exposures and potential confounders	12,29-31
		(b) Indicate number of participants with missing data for each variable of interest	7
		(c) Summarise follow-up time (eg, average and total amount)	13
Outcome data	15	Report numbers of outcome events or summary measures over time	13
Main results	16	(a) Give unadjusted estimates and, if applicable, confounder-adjusted estimates and their precision (eg, 95% confidence interval). Make clear which confounders were adjusted for and why they were included	13,14, 35
		(b) Report category boundaries when continuous variables were categorized	29
		(c) If relevant, consider translating estimates of relative risk into absolute risk for a meaningful time period	12, 33
Other analyses	17	Report other analyses done—eg analyses of subgroups and interactions, and sensitivity analyses	S5-S8
Discussion			
Key results	18	Summarise key results with reference to study objectives	16
Limitations	19	Discuss limitations of the study, taking into account sources of potential bias or imprecision. Discuss both direction and magnitude of any potential bias	19
Interpretation	20	Give a cautious overall interpretation of results considering objectives, limitations, multiplicity of analyses, results from similar studies, and other relevant evidence	18,19
Generalisability	21	Discuss the generalisability (external validity) of the study results	18
Other information			17

Funding	22	Give the source of funding and the role of the funders for the present study and, if applicable, for the original study on which the present article is based	20

Supplemental Table 2. Reclassification table of RTRs.

		Updated Model				
		RTRs in Risk categories (*n*)				
		Low	Medium low	Medium high	High	RTR reclassified (%)
Initial Model	Low	188	19	2	0	10
	Medium low	22	34	10	0	48
	Medium high	7	15	38	13	48
	High	0	2	15	83	17

Supplemental Table 3. Diet components in subjects at the third TMAO tertile.

	Median (IQ range)	Mean (SD)	Population mean consumption[1]
**Egg intake, g/day**	8.92(7.14,14.28)	12.68 (9.9)	12
**Vegetable Intake, g/day**	95.75(65.83,140.15)	106.81 (70.1)	127
**Fruit Intake, g/day**	99.43(52.71,191.41)	121.25 (84.8)	122
**Fish and Seafood Intake, g/day**	14.87(4.679,22.95)	17.242 (16.0)	15

Supplemental Table 4. Comparison of Standardized Net Benefits of two predictive models.

Risk Threshold	Traditional Model	TMAO & Diet enriched Model
0.1	0.646	0.653
0.2	0.466	0.509
0.3	0.333	0.360
0.4	0.247	0.276
0.5	0.155	0.207
0.6	0.103	0.207
0.7	0.017	0.080

Supplemental Figure 1. A

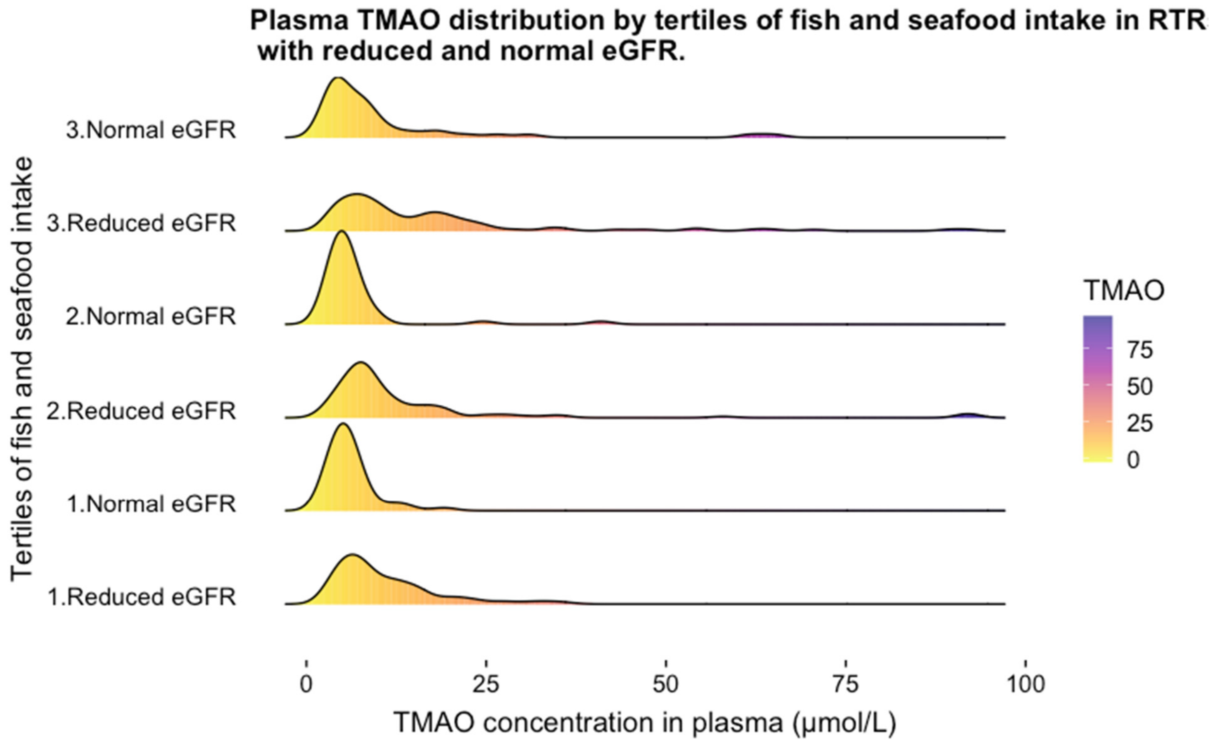

Tertiles of fish and seafood intake in RTRs with reduced eGFR (<60 ml/min * 1.73m^2)

1.Reduced eGFR = 0 – 7.55 g/day
2.Reduced eGFR = 7.55 – 17.1 g/day
3.Reduced eGFR = 17.1 – 106 g/day

Tertiles of fish and seafood intake in RTRs with normal eGFR (>=60 ml/min * 1.73m^2)

1.Normal eGFR = 0 – 4.69 g/day
2.Normal eGFR = 4.69 – 15.7 g/day
3.Normal eGFR = 15.7 – 80.8 g/day

Supplemental Figure 1. B

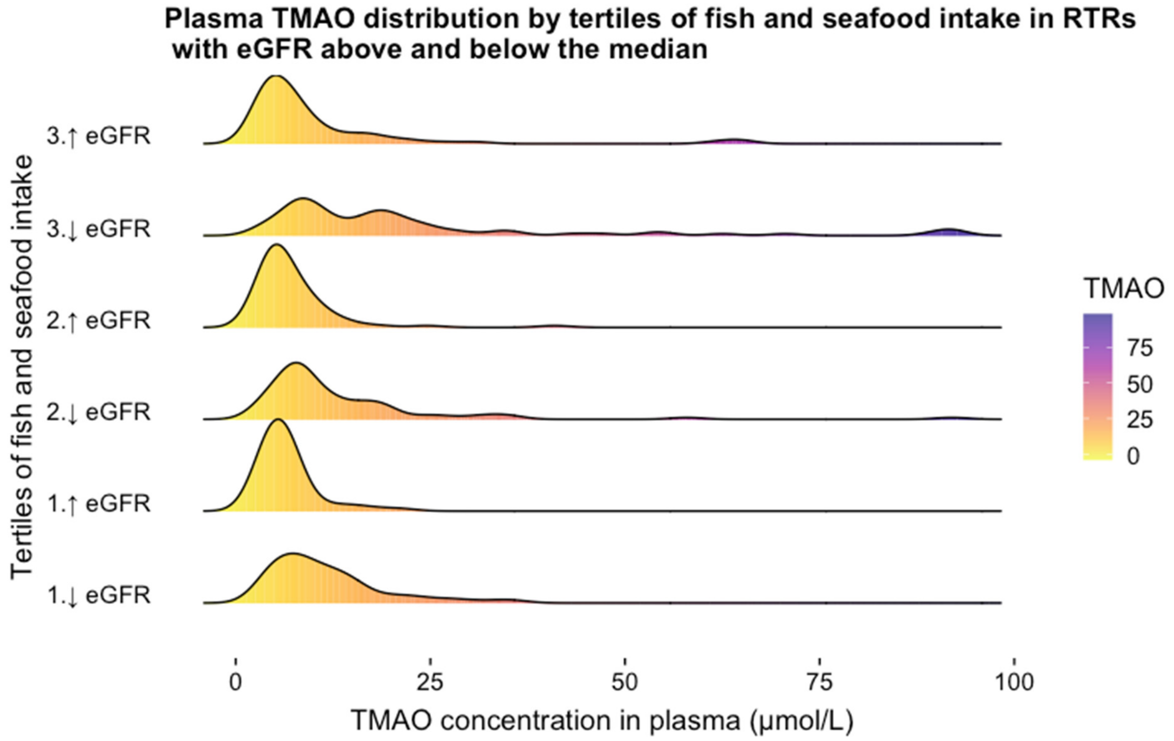

Tertiles of fish and seafood intake in RTRs with eGFR below the median (< 47.96 ml/min * 1.73m^2)

1. ↓ eGFR = 0 – 6.45 g/day
2. ↓ eGFR = 6.45 – 16.7 g/day
3. ↓ eGFR = 16.7 – 106 g/day

Tertiles of fish and seafood intake in RTRs with eGFR above the median (>=47.96 ml/min * 1.73m^2)

1. ↑ eGFR = 0 – 6.97 g/day
2. ↑ eGFR = 6.97 – 16.8 g/day
3. ↑ eGFR = 16.8 – 80.8 g/day

Supplemental Figure 2.A

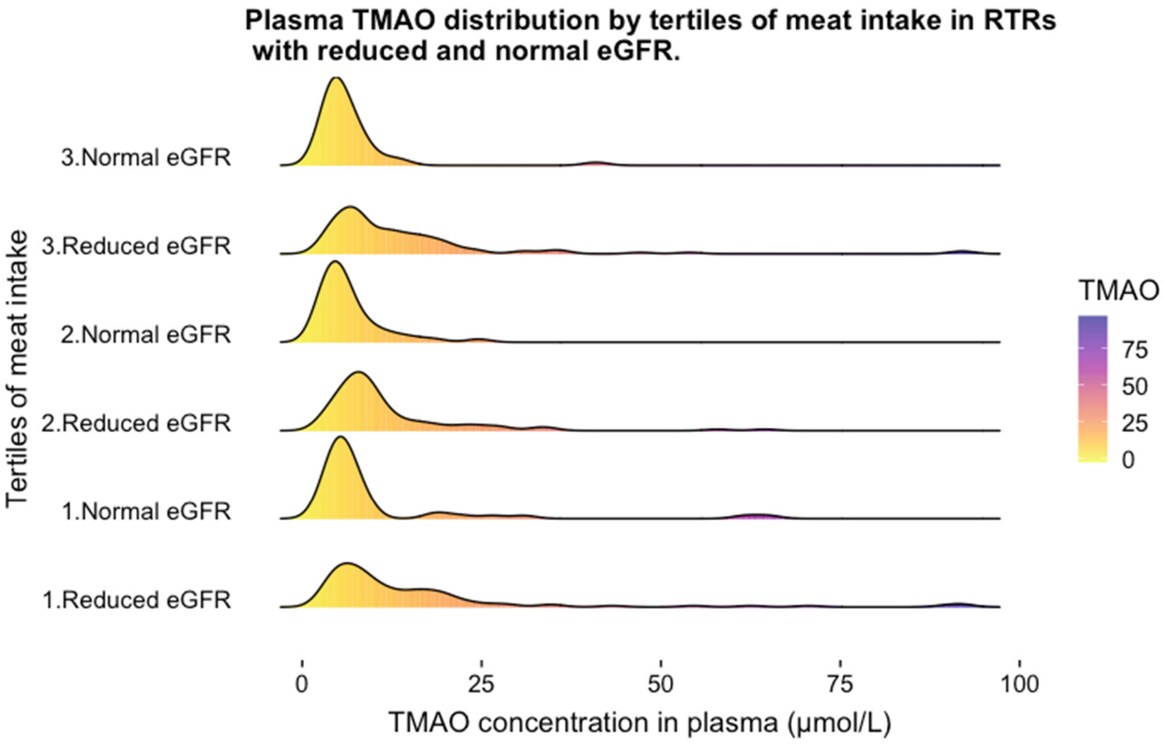

Tertiles of meat intake in RTRs with reduced eGFR (< 60 ml/min * 1.73m^2)

1. Reduced eGFR = 0.1 – 71.4 g/day
2. Reduced eGFR = 71.4 – 93.5 g/day
3. Reduced eGFR = 93.5 – 270 g/day

Tertiles of meat intake in RTRs with normal eGFR (>= 60 ml/min * 1.73m^2)

1. Normal eGFR = 0.1 – 70 g/day
2. Normal eGFR = 70 – 97.4 g/day
3. Normal eGFR = 97.4 – 158 g/day

Supplemental Figure 2.B

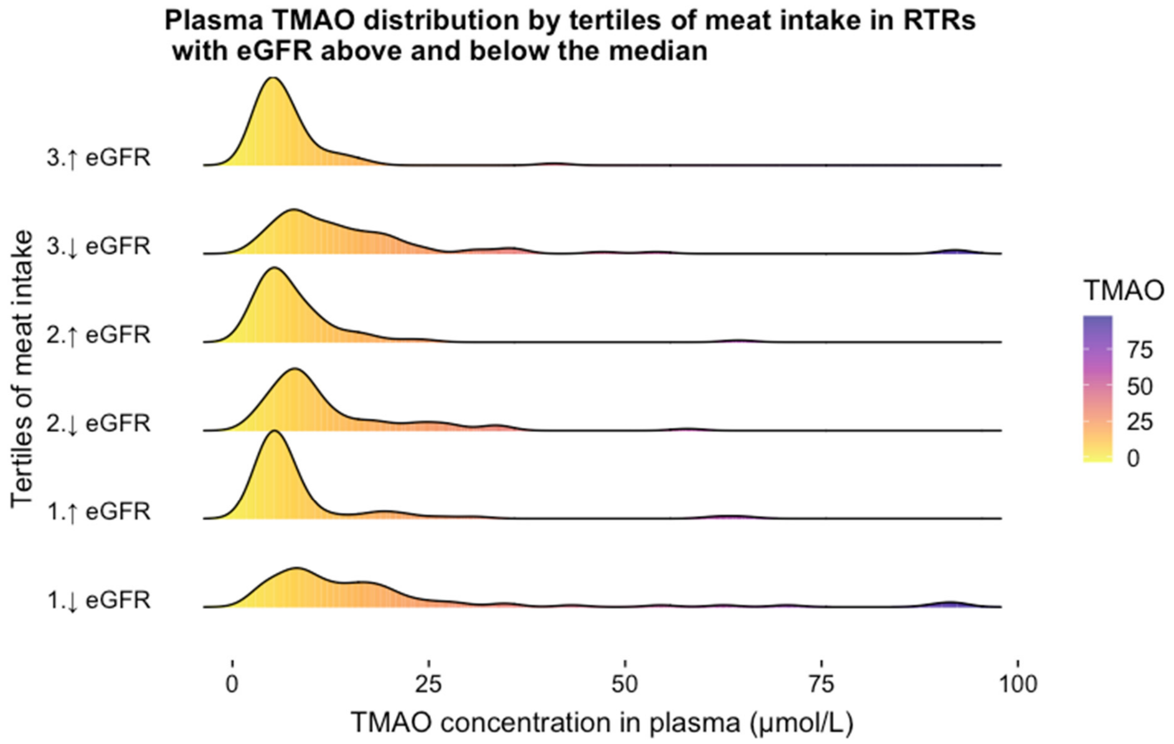

Tertiles of meat intake in RTRs with eGFR below the median (<47.96 ml/min * 1.73m²)

1. ↓ eGFR = 0.1 – 70 g/day
2. ↓ eGFR = 70 – 93.4 g/day
3. ↓ eGFR = 93.4 – 205 g/day

Tertiles of meat intake in RTRs with eGFR below the median (>=47.96 ml/min * 1.73m²)

1. ↑ eGFR = 0.1 – 71.3 g/day
2. ↑ eGFR = 71.3 – 96.1 g/day
3. ↑ eGFR = 96.1 – 270 g/day

Supplemental Figure 3.

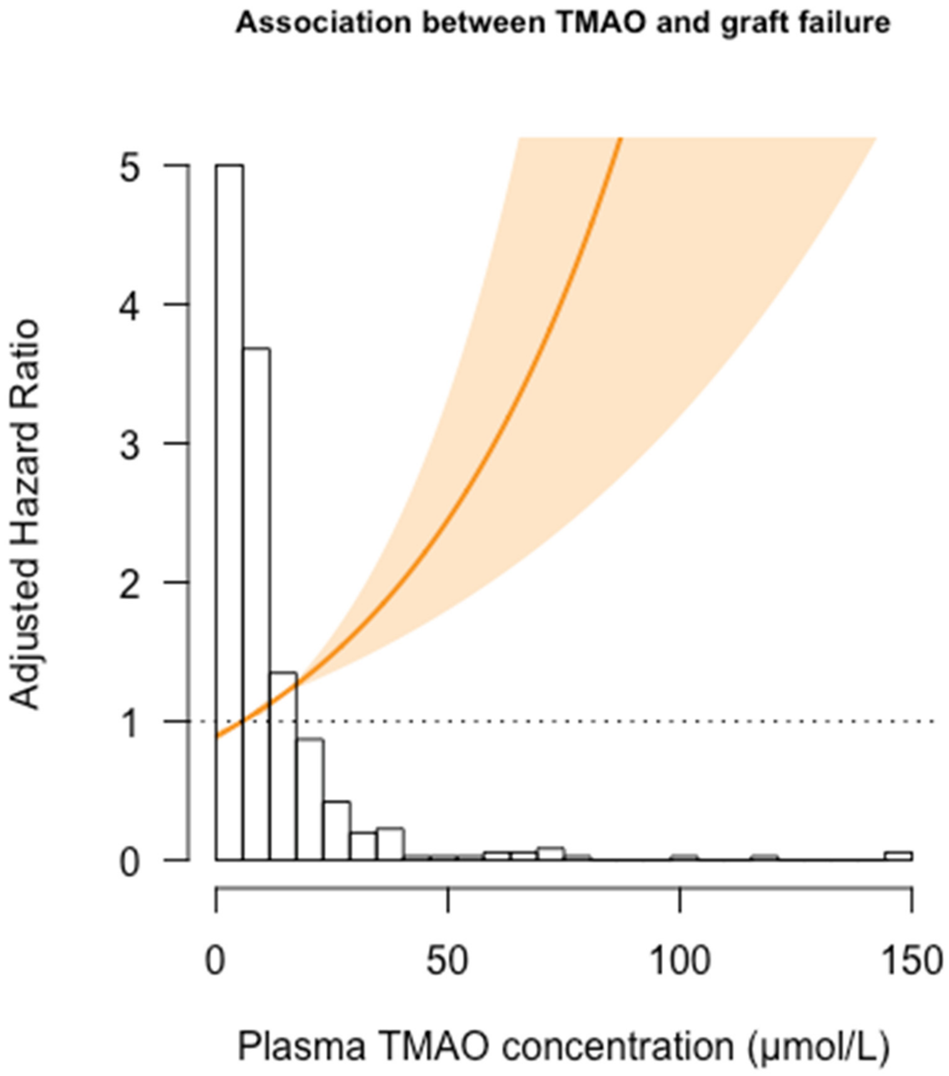

Supplemental References.

1. The diet of the Dutch: Results of the first two years of the Dutch National Food Consumption Survey 2012-2016 | RIVM. https://www.rivm.nl/publicaties/diet-of-dutch-results-of-first-two-years-of-dutch-national-food-consumption-survey-2012. Accessed 22 Dec 2020
